# Potential Toxicity of the Essential Oil from *Minthostachys mollis*: A Medicinal Plant Commonly Used in the Traditional Andean Medicine in Peru

**DOI:** 10.1155/2019/1987935

**Published:** 2019-12-19

**Authors:** Juan Pedro Rojas-Armas, Jorge Luis Arroyo-Acevedo, José Manuel Ortiz-Sánchez, Miriam Palomino-Pacheco, Hugo Jesus Hilario-Vargas, Oscar Herrera-Calderón, Julio Hilario-Vargas

**Affiliations:** ^1^Laboratory of Experimental Pharmacology, Institute of Clinical Research, Faculty of Medicine, Universidad Nacional Mayor de San Marcos, Lima, Peru; ^2^Section of Physiology, Faculty of Medicine, Universidad Nacional Mayor de San Marcos, Lima, Peru; ^3^Section of Biochemistry, Faculty of Medicine, Universidad Nacional Mayor de San Marcos, Lima, Peru; ^4^Laboratory of Pharmacognosy and Traditional Medicine, Faculty of Pharmacy and Biochemistry, Universidad Nacional Mayor de San Marcos, Lima, Peru; ^5^Department of Physiology, School of Medicine, National University of Trujillo, Trujillo, Peru

## Abstract

Medicinal plants are used throughout the world and the World Health Organization supports its use by recommending quality, safety and efficacy. *Minthostachys mollis* is distributed in the Andes of South America and is used by the population for various diseases. While studies have shown their pharmacological properties, the information about their safety is very limited. Then, the goal of this research was to determine the acute oral toxicity and in repeated doses during 28 days of *Minthostachys mollis* essential oil (Mm-EO) in rats. For the acute toxicity test two groups of rats, of three animals each, were used. Each group received Mm-EO in a single dose of 2000 or 300 mg/kg of body weight. For the repeated dose toxicity test, four groups of 10 rats each were used. Doses of 100, 250 and 500 mg/kg/day were used, one group was control. With the single dose of Mm-EO of 2000 mg/kg of body weight, the three rats in the group showed immediate signs of toxicity and died between 36 and 72 hours. In the lung, inflammatory infiltrate was observed, predominantly lymphocytic with severe hemorrhage and presence of macrophages with hemosiderin. In the repeated dose study, male rats (5/5) and female rats (2/5) died at the dose of 500 mg/kg/day. The body weight of both male and female rats decreased significantly with doses of 250 and 500 mg/kg/day. The serum levels of AST and ALT increased significantly and the histopathological study revealed chronic and acute inflammatory infiltrate in the lung; while in the liver was observed in 80% of the cases (24/30) mild chronic inflammatory infiltrate and in some of those cases there was vascular congestion and in one case cytoplasmic vacuolization. The Mm-EO presented moderate acute oral toxicity, while with repeated doses for 28 days; there was evidence of toxicity, in a dose-dependent manner, mainly at the hepatic level.

## 1. Introduction

The genus *Minthostachys* (Benth.) Spach (family Lamiaceae) has 17 species of aromatic shrubs distributed in the Andes of South America [1]. *Minthostachys mollis* (Benth.) Griseb is restricted to the Andes of Venezuela, Colombia, Ecuador, Peru and Bolivia. Is known by various popular names and has different medicinal uses. In Venezuela, *M. mollis* is known as oreganote and is prepared as tea and used to treat infectious diseases of children, as well as for rheumatism [2]. In Peru, the most well-known name of *M. mollis* is “muña”, but in some regions it is also known as “ishmuña”, “tento”, “tinto” and “champca”; Traditionally it is used for the treatment of cough, bronchitis, stomach ulcer, gastritis, stomach and intestinal spasm, cold, headache, intestinal parasitism and as digestive [3–5].

It has been shown that *M. mollis* possesses pharmacological properties. In this regard, the alcoholic extract of this species showed inhibitory activity on *Eschericchia coli* [6]. On the other hand, the essential oil of *M. mollis* had significant inhibitory effect against Gram positive and Gram negative bacteria, especially *Bacillus subtilis* and *Salmonella typhi* [2]. Antifungal effect against *Candida albicans*, and dermatophytes such as *Microsporum canis, Trichophyton mentagrophytes, Trichophyton tonsurans, Trichophyton rubrum * and *Fusarium oxysporum* [7, 8]. As well as cytotoxic activity against prostate cancer cells DU-145 and breast cancer cells MCF-7 [9].

Medicinal plants are widely used throughout the world. Peru is a country rich in biodiversity. Traditional healers have used the rich flora for millennia to cure diseases and until now they are still using the same plants. Traditional medicine continues to be very popular because a large part of the population does not have access to or does not have the economic resources to use Western medicine. This scenario makes it essential to scientifically validate its pharmacological properties and establish the safety of medicinal plants to safeguard the health of the population, as emphasized by the World Health Organization: quality, safety and efficacy [10].

At present there are little information in the scientific literature about the safety of *Minthostachys mollis*, so the purpose of the present research was to evaluate the acute oral toxicity and in repeated doses for 28 days of the essential oil of *Minthostachys mollis* in rats.

## 2. Materials and Methods

### 2.1. Medicinal Plant

The plant *Minthostachys mollis* was obtained in a market in the city of Lima, Peru; a sample was taken to the Natural History Museum of the UNMSM for taxonomic identification (ID No. 185-USM-2015). The essential oil was obtained by steam distillation in a Clevenger type apparatus [11], for which we used fresh leaves. The essential oil was separated and dehydrated with anhydrous sodium sulfate, filtered and stored in an amber glass bottle under refrigeration at a temperature of 4°C until use.

### 2.2. Experimental Animals and Housing

Albino Holtzman rats were obtained from the National Health Institute, Peru. They were kept in their cages for one week prior to commencing the study to allow for acclimatization to the laboratory conditions. Animals housing was maintained under controlled environmental conditions (12 h light/dark cycle) and temperature (22 ± 3°C). They were fed ad libitum with commercial rat feed and drinking water. All the animal experiments were performed in accordance with institutional protocols and the guideline for care and use of laboratory animals [12]. The present research was approved by the Ethics Committee of the School of Medicine, Universidad Nacional Mayor de San Marcos, Peru (ID No. 0262).

### 2.3. Acute Oral Toxicity Studies

Acute toxicity at a single dose was evaluated according to the Organization for Economic Co-operation and Development (OECD) guideline, method 423 [13]. All the animals were fasted overnight before commencing the experiment. Six female rats (160 ± 10 g bw) were used, which were randomly assigned to 2 groups (*n* = 3) that received a single dose of essential oil (300 and 2000 mg/kg of bw). The animals were observed individually during the first 30 minutes; with special attention during the first 4 hours and daily until the 14 days of the experiment. Signs and symptoms of toxicity were written down. The observations were directed to the determination of: Death and time of occurrence, signs and symptoms of toxicity including beginning and duration. In addition to changes in the skin, fur, mucous membranes and eyes, respiratory and circulatory system, central nervous and autonomic, somatomotor activity and behavior. Special attention was paid to the potential occurrence of tremor, seizures, salivation, diarrhea, lethargy, drowsiness and coma. To conclude the experiment, animals were sacrificed by inhalation of ethyl ether, and then were performed necropsy and macroscopic pathological study of the stomach, liver, spleen, lungs, kidneys, esophagus, brain, and small intestine. Finally, the organs were studied by microscopic examination.

### 2.4. Repeated Dose 28-Days Oral Toxicity Study

The sub-chronic oral toxicity was performed in accordance with the instructions by OECD test guidelines-407 [14] with slight modifications. Twenty female rats (160 ± 10 g bw) and twenty male rats (170 ± 10 g bw) were used. Were randomly assigned to 4 groups (*n* = 10: five female and five male). Each rat of group I received only vehicle (control group). Groups II, III, and IV received the *Minthostachys mollis* essential oil (Mm-EO) in repeated oral doses of 100, 250, and 500 mg/kg bw, respectively, for 28 days. Animals were dosed at approximately the same time each day. The animals were monitored for signs of toxicity and mortality twice a day (a.m. and p.m.) throughout the experimental period of 28 days. The terminal weight of each animal was recorded weekly throughout the course of the experiment. On day 29, blood samples were collected by intracardiac puncture in the rats under anesthesia with ethyl ether for the assessment of haematological and biochemical parameters.

The animals were sacrificed by decapitation under anesthesia with intraperitoneal sodium pentobarbital (40 mg/kg). Organ were fixed in 10% formalin for histopathological examination.

### 2.5. Body and Organ Weight Measurement

After treatments, were measured and recorder the body weight of each rat in the experiment. The heart, lungs, liver, spleen, stomach, kidney, testis and uterus were excised immediately after the sacrifice, trimmed of fat and connective tissue, blotted with filter paper and weighed. The relative organ weights [ratio of organ weight and the animal's body weight (at the end of experiment) × 100] were calculated.

### 2.6. Biochemical Parameters

Were evaluated in a Semi-Automatic Biochemical Analyzer model EMP-168 (Ivdiagnostik®, Emperor Medical) according to the manufacturer's specifications. The levels of aspartate aminotransferase (AST), alanine aminotransferase (ALT), alkaline phosphatase, total protein, bilirubin, cholesterol, triglycerides, high-density lipoprotein (HDL), low-density lipoprotein (LDL), total albumin, glucose, urea and creatinine were determined.

### 2.7. Hematological Assay

Were performed in an Automatic Hematology Analyzer KT-6400 (Genius®, Med Equipment). At the end of the experiment were evaluated: Hematocrit, hemoglobin concentration, erythrocyte count, total and differential leukocyte count, and platelet count.

### 2.8. Histopathological Analysis

At the end of oral toxicity study, all the animals were subjected to necropsy or may be earlier in case of death. The organs were preserved in 10% formalin and fixed for 3 days, dehydrated, embedded in paraffin, sectioned at 5 *μ*m and stained with hematoxylin/eosin. Slides of organs taken from all animals were examined microscopically and photographed with a light microscope (Olympus BX53, Tokyo, Japan) at 100x and 400x magnification.

### 2.9. Statistical Analysis

The data were expressed as mean ± S.D of five animals in each group and were analyzed by one-way analysis of variance (ANOVA) followed by Tukey test. The results were considered significant when *p* < 0.05. The statistical computer software program, SPSS version 19 was used.

## 3. Results

### 3.1. Acute Oral Toxicity

With the single dose of Mm-EO of 2000 mg/kg of body weight, three rats in the group showed rapid signs of toxicity; included hyperactivity, irritation, tingling and stinging of the nose. Followed by ataxia and depression in 2 hours, then went into torpor and died between 36 and 72 hours. Faced with these results, we proceeded according to the stipulations of guideline 423 of the OECD and a single dose of 300 mg/kg of Mm-EO was administered to another group of rats; all 3 animals showed no sign of toxicity.

**Figure 1:**
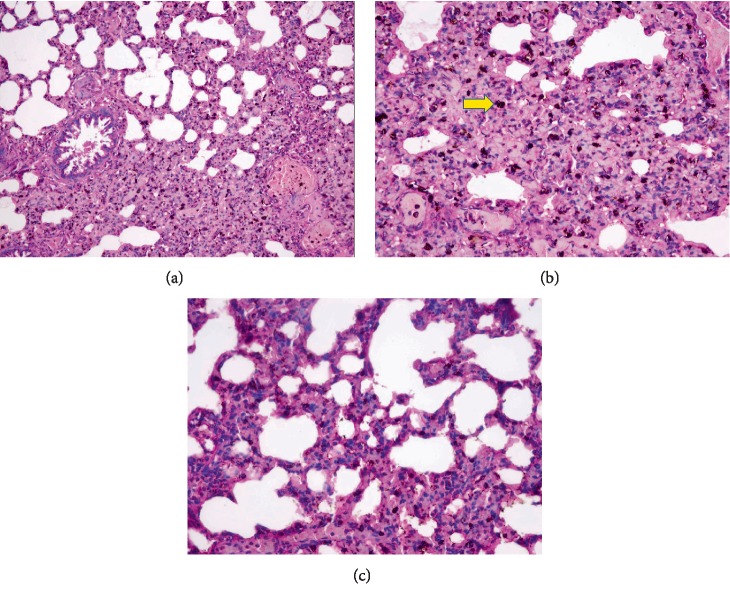
Lung tissue from rats that received a single dose, 2000 mg/kg of Mm-EO. (a) Abundant erythrocytes and erythrocyte residues. (b) The arrow indicates the presence of macrophages with hemosiderin. (c) Abundant inflammatory infiltrate. H&E stain and 400X.

In the macroscopic examination of organs from dead rats that received Mm-EO at the maximum dose of 2000 mg/kg of body weight, diffuse haemorrhagic congestion was observed only in lungs. Microscopic examination in lung showed inflammatory infiltrate, predominant lymphocytic infiltration with severe hemorrhage and the presence of hemosiderin macrophages (Figure 1). In other organs, no alterations were observed.

### 3.2. Repeated Dose 28-Days Oral Toxicity Studies

In the group of male rats, all animals that received Mm-EO in doses 500 mg/kg/day (5/5) died between 6 and 11 days. In the group of female rats there was, also, mortality with the dose of 500 mg/kg/day but just two animals (2/5). The first died on day 12 and the second, on day 24. All animals of the groups that received Mm-EO at a dose of 100 mg/kg/day and 250 mg/kg/day survived during 28-day treatment period.

### 3.3. Effect of Essential Oil on Rat's Body and Organ Weight

**Figure 2:**
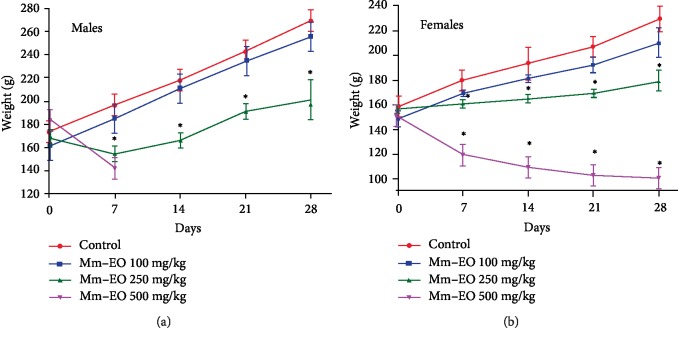
Body weight in rats treated with *Minthostachys mollis* essential oil (Mm-EO) repeated oral doses (100, 250, and 500 mg/kg) for 28 days. ^∗^*p* < 0.05 compared to the control.

The body weight of the male rats that received treatment with Mm-EO at a dose of 500 mg/kg/day drastically decreased at the end of the first week to 142.3 ± 10.1 g compared to 196.7 ± 10.4 g of the control group (*p* < 0.05); which culminated in the death of all the animals in this group. With the dose of 250 mg/kg/day there was a significant decrease in body weight from the first week until the end of the experiment where 201.7 ± 18.2 g was showed compared to 269.7 ± 7.6 g of the control group (*p* < 0.05). In female rats there was a significant decrease in body weight with the doses of 250 and 500 mg/kg/day from the first week of treatment with Mm-EO until the fourth week ended; being more pronounced with the group that received 500 mg/kg/day where 100.5 ± 3.5 g was recorded compared to 229.0 ± 10.6 g of the control group (*p* < 0.05). With the dose of 100 mg/kg/day, there was no significant weight variation in both male and female rats (Figure 2).

The liver relative weight increased significantly (*p* < 0.05) in the group treated with Mm-EO at a dose of 250 mg/kg/day in the 28-day period, in both males and females. With the dose of 500 mg/kg/day, the relative weight of heart, lungs, liver, stomach and testicles or uterus increased gnificantly (*p* < 0.05) compared to the control group (Table 1).

### 3.4. Effect of Essential Oil on Biochemical Parameters

At the end of the 28-day treatment period, serum hepatic enzyme levels were significantly increased in male rats receiving Mm-EO 250 mg/kg/day, thus the AST ranged from 144.33 ± 6.81 IU/L (control) to 171.33 ± 11.37 IU/L (*p* < 0.05); while ALT ranged from 61.67 ± 13.61 IU/L (control) to 99.00 ± 11.53 IU/L (*p* < 0.05). These enzymes also increased significantly in female rats, ALT with the dose of 250 mg/kg/day, and both ALT and AST with the dose of 500 mg/kg/day. Likewise, a significant decrease in serum creatinine was observed in both male and female rats. On the other hand, there was a surprisingly significant decrease in blood levels of cholesterol, triglycerides and LDL, both in males and females, with a dose of 250 and 500 mg/kg/day (Table 2).

### 3.5. Effect of Essential Oil on Haematological Parameters

Results of hematological parameters of control rats and those treated daily for 28 days with the Mm-EO are showed in Table 3. These results show that only neutrophils varied significantly with respect to the control group, increasing from 19.00 ± 1.53 (control) to 27.33 ± 2.52 (*p* < 0.05) in female rats; whereas neutrophs were also increased in male rats, but the difference was not significant.

### 3.6. Histological Findings

**Figure 3:**
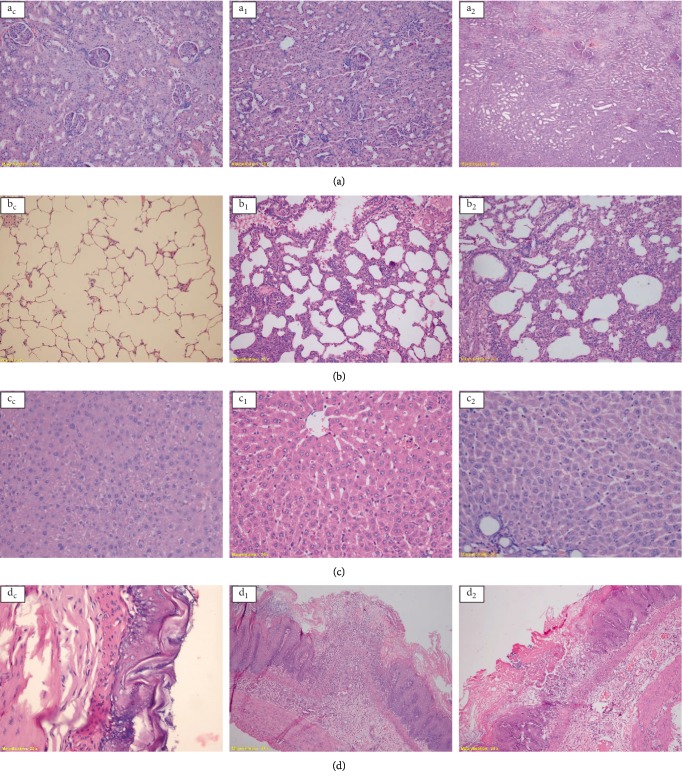
Photomicrographs of the sections from kidney (a), lung (b), liver (c), and esophagus (d) of rats treated with *Minthostachys mollis* essential oil during 28 days. Subindices C, 1 and 2 refer to the control groups, 250 and 500 mg/kg/day, respectively. H-E staining, 40X.

Microscopic examination of the organs at the end of the 28-day treatment period with the Mm-EO showed that the spleen did not suffer alterations with any of the doses used, whereas in the kidney only with the dose of 500 mg/kg/day observed a case (1/10) of severe tubular necrosis, although the glomeruli were intact (Figure 3a_2_). In lung severe chronic and acute inflammatory infiltrate was observed in 100% of the cases with the 3 dose levels, of which, in addition in two cases (2/30) hemorrhagic foci and necrosis were observed with the dose of 500 mg/kg/day (Figure 3b_2_). In the liver, with the three dose levels, 80% of the cases (24/30) had a mild chronic inflammatory infiltrate (Figures 3c_1_ and 3c_2_), and in some of these cases there was also vascular congestion and in one case cytoplasmic vacuolization. In stomach only in two cases (7% of all cases) mild acute inflammatory infiltrate was observed. In esophagus, in 10% of cases (3/30) acute ulcerated esophagitis was observed (Figures 3d_1_ and 3d_2_).

## 4. Discussion

The manifestations of toxicity after receiving the maximum oral dose of 2000 mg/kg of Mm-EO were quickly demonstrated at the level of the respiratory system and the central nervous system (CNS). The stinging of the nose could be due to the Mm-EO, by its volatile nature, after being absorbed in the gastrointestinal tract a part follows this path to elimination where it causes sensory irritation. This irritation can be caused by the stimulation of the receptors in the trigeminal nerves [15]. However, the toxic effect would be mainly located at the level of the CNS where hyperexcitability was initially observed and then evolved into lethargy, coma and death. The CNS is particularly vulnerable to toxic substances because it has very limited capacity to regenerate, and if the nerves are damaged cannot recover; causing behavioral changes and muscle effects such as weakness, numbness and alterations in motor coordination. Thus, both CNS stimulation and depression are manifestations of functional neurotoxicity [16].

In a previous study, we determined by GC–MS that the main components of Mm-EO were pulegone (33.48%) and menthone (26.68%) [17]. The effects of Mm-EO observed in the acute oral test would be related to these two components. Pulegone is rapidly and extensively absorbed from the gastrointestinal tract after the single oral administration and was found mainly in the liver, kidney, blood and lung after oral administration [18], which would indicate that the manifestations of toxicity at the level of the airway would be partly due to pulegone. Likewise, it has been reported that both menthone and pulegone, without acting directly on the dopamine receptors, change the levels of extracellular dopamine, promoting ambulation in mice and in high doses producing ataxia [19, 20]. This could explain the initial hyperactivity after the administration of Mm-EO and the subsequent ataxia until the death of the animals occurred.

In the rats' lung tissue that were subjected to the acute oral test with Mm-EO (Figure 1) abundant erythrocytes and erythrocytes remains were observed, which would indicate the presence of hemorrhage; macrophages were also seen with hemosiderin, which would be the result of phagocytosis of red blood cells and release of iron from the heme group. Likewise, there were abundant inflammatory infiltrates in the alveolar septa, congruent with pulmonary capillaritis, which would produce necrosis of these structures and lead to a gradual loss of capillary structural integrity. Thus, allowing extravasations of red blood cells to the alveolar space and to the pulmonary interstitium. In addition, most of the neutrophils, when fragmented, release oxygen free radicals and proteolytic enzymes into the alveolar spaces and interstitium, producing lung injury. This pulmonary toxic effect is mainly due to pulegone, which is volatile and uses the lung as one of the routes for its elimination. This is because the pulegone has a vapor pressure of 138 mmHg at 25°C [21]. In a single dose toxicity study of pennyroyal oil, where pulegone was the main component with approximately 90% of the total oil, in mice (400 mg/kg ip), bronchial epithelial necrosis was found in addition to liver necrosis [22].

Oral administration of Mm-EO in repeated doses during 28 days produced some manifestations of toxicity, according to the results of survival throughout the study, the histological, hematological, biochemical and body weight control tests as well as the relative weight of the organs. The Mm-EO at doses of 100 and 250 mg/kg/day did not produce mortality of the animals during the study; whereas the dose of 500 mg/kg/day caused the death of all the male rats of this group (5/5) in the early stage of the experiment (6–11 days), and of 2 female rats (2/5) in the period of 12–24 days. This lethal effect could be mainly due to pulegone, given that a study with pulegone 300 mg/kg reported survival in male 0/5 rats and in females 1/5 and early deaths occurred on or before day 5 of the experiment [23].

At the hepatic level, the histopathological study revealed a mild chronic inflammatory infiltrate in 80% of the cases (Figures 3c_1_ and 3c_2_) and in some of those cases there was also vascular congestion, as well as cytoplasmic vacuolization in one case. In addition, serum levels of aspartate aminotransferase (AST) and alanine aminotransferase (ALT) enzymes increased significantly, while alkaline phosphatase and bilirubin also increased, although not significantly (Table 2). AST and ALT are sensitive indicators of hepatocellular damage [24] and it is claimed that even a small increase in bilirubin means the possibility of liver damage [25]. Added to this is the significant increase in the relative weight of the liver (Table 1), which could be due to inflammation. Taken together, these results could indicate a probable liver damage, whose effect would be dependent on the dose of Mm-EO. There are several studies who show that pulegone is hepatotoxic when given in repeated doses. Administration of pulegone 400 mg/kg/day to rats resulted in destruction of liver Cit.P-450 both *in vivo* and *in vitro* [26]. In another similar study, pulegone caused significant decrease in hepatic microsomal Cit-P450 and hem, as well as massive hepatotoxicity accompanied by an increase in serum TGP (ALT) [27]. Pulegone orally administered to rats at a dose of 160 mg/kg/day for 28 days increased the levels of plasma alkaline phosphatase and the relative weight of the liver indicating an adverse effect on the liver, although the histopathology of the liver was not significant [28]. It has also been reported that menthone administered orally to rats for 28 days produced an increase in the relative weight of the liver and an increase in the activity of alkaline phosphatase and plasma bilirubin, although these changes were not accompanied by microscopic changes in the liver [29]. On the other hand, pulegone was identified as the main hepatotoxic component of pennyroyal oil, causing centrilobular hepatic necrosis in mice and rats [30]. In this sense, both pulegone and menthone would be responsible for the hepatotoxic effect of Mm-EO.

The organ that was affected by the repeated administration of Mm-EO, in all cases and with the 3 dose levels, was the lung. Severe chronic and acute inflammatory infiltrate was observed, and with the dose of 500 mg/kg/day two hemorrhagic foci and necrosis were observed in two cases (Figure 3b_2_); with this same dose, a significant increase in neutrophils was observed (Table 3). Probably this effect is also caused by the pulegone that uses the pulmonary route for its elimination, generating inflammation and with the highest dose, tissue injury. At the renal level, severe tubular necrosis with intact glomeruli (Figure 3a_2_) only occurred in one case. A study reported that pulegone produced a single case of an unusual renal lesion [23]. Ulcerated acute esophagitis was observed in 10% of the cases (Figures 3d_1_ and 3d_2_), which could be due to mechanical injury produced by the orogastric tube used to administer Mm-EO.

In this investigation was observed that the body weight of the animals treated with Mm-EO decreased significantly, in a dose-dependent manner, from the first week of treatment (Figure 2); likewise, serum creatinine values decreased significantly (Table 2). Another study reported that pulegone administered in repeated doses for 28 days, caused a significant decrease in the body weight of the rats from the first week of treatment, accompanied by a decrease in creatinine [28]. The loss of body weight is an important indicator of toxicity because it reflects a series of organic changes in individuals, while the decrease in serum creatinine is related to the reduction of skeletal muscle mass [31].

The toxicity of pulegone is widely attributed to its metabolism to reactive species, in particular menthofuran, which can undergo rearrangement to electrophilic *γ*-ketone species; reactive metabolites adduce cellular macromolecules and deplete glutathione levels leading to toxicity [32]. *In vitro* studies in human liver microsomes have shown the participation of CYP-450 enzymes, mainly CYP2E1, in the metabolism of pulegone where its main metabolite is menthofuran which is more toxic than pulegone. These findings were confirmed by *in vivo* studies in which the direct relationship between the metabolism of pulegone by CYP-450 and hepatotoxicity was demonstrated. Thus, menthofuran produced an 88% decrease in the activity of mitochondrial aldehyde dehydrogenase and a 34% decrease in the activity of the complex V of mitochondrial ATP synthase [30].

An extensive study of pulegone toxicity, where it was administered chronically for a period of 2 years, showed urinary bladder cancer in female rats and liver cancer in mice, a slight increase in rare bone lesions such as osteoma and osteosarcoma, as well as nonneoplastic lesions in the nose of rats and mice [23]. Another study demonstrated that pulegone administered in high doses is excreted and concentrated in the urine at cytotoxic levels where it produced extensive epithelial cell necrosis and urothelial tumors in rats [33].

Currently there is no previous report of oral toxicity of Mm-EO. Based on the results obtained in the oral acute toxicity test, as established by the OECD according to the Globally Harmonized Classification System (GHS), the Mm-EO belongs to category 4 (>300–2000 mg/kg of body weight), with a cut-off point of LD_50 _= 500 mg/kg of body weight [13]. This result, according to Tisserand & Young, 2013 [16], is classified as moderately toxic. The results of the oral toxicity test in repeated doses during 28 days suggest that the Mm-EO produces important dose-dependent toxicity, mainly in the liver.

## 5. Conclusions

Under conditions of this experiment, Mm-EO presents moderate acute oral toxicity with an LD_50 _= 500 mg/kg of body weight. While with repeated doses for 28 days, there was evidence of toxicity, in a dose-dependent manner, mainly at liver level, which would be related to pulegone and menthone which are the most abundant components of Mm-EO.

## Figures and Tables

**Table 1:** Effect of *Minthostachys mollis* essential oil (Mm-EO) on the relative weight of the organs in treated rats for 28 days.

Organ	Control	Mm-EO 100	Mm-EO 250	Mm-EO 500
*Males*				
Heart	0.32 ± 0.01	0.33 ± 0.05	0.36 ± 0.09	0.49 ± 0.07^∗^
Lungs	0.70 ± 0.12	0.73 ± 0.14	0.88 ± 0.14	1.10 ± 0.15^∗^
Liver	3.10 ± 0.17	3.21 ± 0.10	3.79 ± 0.09^∗^	4.90 ± 0.30^∗^
Spleen	0.35 ± 0.04	0.38 ± 0.11	0.41 ± 0.08	0.48 ± 0.04
Stomach	0.52 ± 0.08	0.55 ± 0.21	0.68 ± 0.29	1.12 ± 0.18^∗^
Kidney	0.37 ± 0.07	0.39 ± 0.09	0.46 ± 0.06	0.55 ± 0.11
Testis	0.92 ± 0.12	0.96 ± 0.14	1.20 ± 0.30	1.63 ± 0.11^∗^

*Females*				
Heart	0.32 ± 0.11	0.35 ± 0,09	0.41 ± 0.14	0.73 ± 0.11^∗^
Lungs	0.95 ± 0.09	1.09 ± 0.14	1.25 ± 0.17	1.95 ± 0.21^∗^
Liver	2.90 ± 0.27	3.10 ± 0.11	3.73 ± 0.15^∗^	4.96 ± 0.23^∗^
Spleen	0.39 ± 0.10	0.42 ± 0.08	0.45 ± 0.12	0.68 ± 0.23
Stomach	0.50 ± 0.07	0.63 ± 0.15	0.82 ± 0.21	1.07 ± 0.27^∗^
Kidney	0.32 ± 0.04	0.34 ± 0.05	0.41 ± 0.10	0.66 ± 0.11^∗^
Uterus	0.47 ± 0.05	0.50 ± 0.10	0.59 ± 0.14	0.91 ± 0.09^∗^

Values are expressed as Mean ± SD, significance when compared with control ^∗^(*p* < 0.05). One-way ANOVA followed by Tukey test.

**Table 2:** Biochemical parameters of rats after treatment with repeated oral doses of *Minthostachys mollis* essential oil (Mm-EO) for 28 days.

Parameters	Control	Mm-EO 100	Mm-EO 250	Mm-EO 500
*Males*				
AST (IU/L)	144.33 ± 6.81	161.67 ± 9.07	171.33 ± 11.37^∗^	n.d.
ALT (IU/L)	61.67 ± 13.61	80.33 ± 10.02	99.00 ± 11.53^∗^	n.d.
Alkaline phosphatase (IU/L)	175.67 ± 9.29	176.33 ± 4.04	181.00 ± 11.53	n.d.
Total bilirubin (mg/dL)	0.61 ± 0.13	0.62 ± 0.07	0.65 ± 0.06	n.d.
Total protein (g/dL)	7.23 ± 0.61	7.33 ± 0.61	7.53 ± 0.32	n.d.
Albumin (g/dL)	3.70 ± 0.36	3.90 ± 0.26	3.83 ± 0.49	n.d.
Cholesterol (mg/dL)	69.83 ± 4.25	65.40 ± 9.18	50.67 ± 8.33^∗^	n.d.
Triglycerides (mg/dL)	102.00 ± 11.79	79.33 ± 13.01	41.00 ± 8.89^∗^	n.d.
HDL (mg/dL)	30.77 ± 4.68	34.00 ± 5.14	35.13 ± 3.56	n.d.
LDL (mg/dL)	18.66 ± 2.55	15.53 ± 3.22	7.34 ± 2.19^∗^	n.d.
Glucose (mg/dL)	116.10 ± 8.65	108.43 ± 11.76	112.17 ± 7.52	n.d.
Urea (mg/dL)	34.00 ± 4.36	33.50 ± 3.12	29.83 ± 1.61	n.d.
Creatinine (mg/dL)	0.94 ± 0.08	0.85 ± 0.08	0.65 ± 0.04^∗^	n.d.

*Females*				
AST (IU/L)	130.33 ± 5.50	144.33 ± 15.63	155.67 ± 12.89	168.33 ± 5.51^∗^
ALT (IU/L)	58.67 ± 9.71	83.67 ± 18.50	101.33 ± 11.37^∗^	109.00 ± 6.56^∗^
Alkaline phosphatase (IU/L)	174.00 ± 9.64	178.67 ± 17.61	177.67 ± 14.19	179.33 ± 6.03
Total bilirubin (mg/dL)	0.62 ± 0.14	0.62 ± 0.11	0.63 ± 0.15	0.66 ± 0.05
Total protein (g/dL)	7.23 ± 0.66	7.50 ± 0.36	7.20 ± 0.65	7.37 ± 0.49
Albumin (g/dL)	3.80 ± 0.20	3.73 ± 0.35	3.67 ± 0.31	3.65 ± 0.04
Cholesterol (mg/dL)	78.40 ± 10.05	58.00 ± 5.07^∗^	45.27 ± 5.00^∗^	42.90 ± 3.73^∗^
Triglycerides (mg/dL)	95.27 ± 11.08	82.03 ± 4.21	31.33 ± 3.21^∗^	30.13 ± 2.80^∗^
HDL (mg/dL)	33.13 ± 4.27	34.00 ± 5.60	34.03 ± 4.59	32.43 ± 2.89
LDL (mg/dL)	26.22 ± 5.15	7.59 ± 2.27^∗^	4.97 ± 0.73^∗^	4.44 ± 1.02^∗^
Glucose (mg/dL)	119.23 ± 6.49	113.23 ± 9.87	110.27 ± 14.14	115.07 ± 3.58
Urea (mg/dL)	35.97 ± 2.34	31.53 ± 2.84	28.47 ± 4.05	32.53 ± 5.06
Creatinine (mg/dL)	0.91 ± 0.08	0.82 ± 0.07	0.63 ± 0.06^∗^	0.60 ± 0.04^∗^

Values are expressed as Mean ± SD, significance when compared with control ^∗^(*p* < 0.05). One-way ANOVA followed by Tukey test. AST, Aspartate aminotransferase; ALT, alanine aminotransferase; ALP, alkaline phosphatase; HDL, high-density lipoprotein; LDL, low-density lipoprotein. n.d., not determined because rats died in the first week of treatment.

**Table 3:** Haematological parameters of rats after treatment with repeated oral doses of* Minthostachys mollis* essential oil (Mm-EO) for 28 days.

Parameters	Control	Mm-EO 100	Mm-EO 250	Mm-EO 500
*Males*				
RBC (×10^6^/*µ*L)	7.22 ± 0.17	7.42 ± 0.22	7.17 ± 0.29	n.d.
WBC (×10^3^/*µ*L)	8.10 ± 0.66	8.43 ± 0.85	8.50 ± 0.82	n.d.
Hemoglobin (g/dL)	14.73 ± 0.85	14.47 ± 0.47	14.33 ± 0.67	n.d.
Hematocrit (%)	46.67 ± 2.08	46.33 ± 3.55	45.97 ± 1.29	n.d.
Neutrophils (%)	18.67 ± 1.53	23.33 ± 3.51	24.67 ± 4.51	n.d.
Eosinophils (%)	2.67 ± 0.58	2.33 ± 0.58	2.67 ± 1.15	n.d.
Basophils (%)	1.33 ± 1.53	1.00 ± 1.00	1.00 ± 1.00	n.d.
Monocytes (%)	3.33 ± 0.58	3.00 ± 1.15	3.00 ± 1.00	n.d.
Lymphocytes (%)	74.00 ± 3.60	67.33 ± 6.56	68.67 ± 5.86	n.d.
Platelets (×10^3^/*µ*L)	707.33 ± 42.45	716.67 ± 35.11	748.33 ± 50.58	n.d.

*Females*				
RBC (×10^6^/*µ*L)	7.17 ± 0.29	6.95 ± 0.25	6.91 ± 0.19	7.05 ± 0.17
WBC (×10^3^/*µ*L)	8.03 ± 0.45	8.73 ± 0.67	10.13 ± 2.95	10.26 ± 2.15
Hemoglobin (g/dL)	13.83 ± 1.89	13.67 ± 1.04	13.60 ± 1.65	13.47 ± 0.65
Hematocrit (%)	42.63 ± 5.73	42.53 ± 3.72	42.33 ± 3.49	42.23 ± 2.11
Neutrophils (%)	19.00 ± 1.53	23.67 ± 4.16	24.67 ± 4.16	27.33 ± 2.52^∗^
Eosinophils (%)	2.33 ± 0.58	1.67 ± 1.52	2.00 ± 1.00	2.00 ± 1.00
Basophils (%)	1.00 ± 1.00	0.67 ± 1.15	1.00 ± 1.00	0.67 ± 0.58
Monocytes (%)	2.67 ± 1.15	2.00 ± 1.00	2.67 ± 2.08	2.33 ± 0.58
Lymphocytes (%)	75.00 ± 2.64	72.00 ± 6.24	69.67 ± 6.51	67.67 ± 4.04
Platelets (×10^3^/*µ*L)	696.33 ± 26.95	747.00 ± 43.97	690.00 ± 29.31	738.33 ± 15.01

Values are expressed as Mean ± SD, significance when compared with control ^∗^(*p* < 0.05). One-way ANOVA followed by Tukey test. RBC, Red blood cell; WBC, White blood cell. n.d., not determined because rats died in the first week of treatment.

## Data Availability

The data used to support the findings of this study are available from the corresponding author upon request.
