# Corrigendum to “Potential Toxicity of the Essential Oil from *Minthostachys mollis*: A Medicinal Plant Commonly Used in the Traditional Andean Medicine in Peru”

**DOI:** 10.1155/2020/2103456

**Published:** 2020-12-03

**Authors:** Juan Pedro Rojas-Armas, Jorge Luis Arroyo-Acevedo, José Manuel Ortiz-Sánchez, Miriam Palomino-Pacheco, Hugo Jesus Justil‐Guerrero, Oscar Herrera-Calderón, Julio Hilario-Vargas

**Affiliations:** ^1^Laboratory of Experimental Pharmacology, Institute of Clinical Research, Faculty of Medicine, Universidad Nacional Mayor de San Marcos, Lima, Peru; ^2^Section of Physiology, Faculty of Medicine, Universidad Nacional Mayor de San Marcos, Lima, Peru; ^3^Section of Biochemistry, Faculty of Medicine, Universidad Nacional Mayor de San Marcos, Lima, Peru; ^4^Laboratory of Pharmacognosy and Traditional Medicine, Faculty of Pharmacy and Biochemistry, Universidad Nacional Mayor de San Marcos, Lima, Peru; ^5^Department of Physiology, School of Medicine, National University of Trujillo, Trujillo, Peru

In the article titled “Potential Toxicity of the Essential Oil from *Minthostachys mollis*: A Medicinal Plant Commonly Used in the Traditional Andean Medicine in Peru” [1], there was a spelling error in the name of the author “Hugo Jesus Justil Guerrero” in the author list, where “Hugo Jesus Hilario-Vargas” should have read as “Hugo Jesus Justil Guerrero.” This is corrected as shown above.
